# An Integrated Analysis of Dostarlimab Immunogenicity

**DOI:** 10.1208/s12248-021-00624-7

**Published:** 2021-07-29

**Authors:** Sharon Lu, Ronald R. Bowsher, Amanda Clancy, Amy Rosen, Mingxuan Zhang, Ying Yang, Kathleen Koeck, Minggeng Gao, Elizabeth Potocka, Wei Guo, Kai Yu Jen, Ellie Im, Ashley Milton

**Affiliations:** 1 Scholar Rock, 301 Binney St 3rd floor, Cambridge, Massachusetts 02142 USA; 2 B2S Life Sciences, Franklin, Indiana, USA; 3 GlaxoSmithKline, Waltham, Massachusetts USA; 4 Certara, Princeton, New Jersey USA; 5 Nuventra Pharma Sciences, Durham, North Carolina USA; 6 Mersana Therapeutics, Cambridge, Massachusetts USA

**Keywords:** Antibody, Immuno-oncology, Immunogenicity, PD-1

## Abstract

Monoclonal antibodies that block the interaction between programmed cell death 1 (PD-1) and its ligand (PD-L1) have revolutionized cancer immunotherapy. However, immunogenic responses to these new therapies—such as the development of antidrug antibodies (ADAs) and neutralizing antibodies (NAbs)—may represent a significant challenge to both efficacy and safety in some patients. Dostarlimab (TSR-042) is an approved, humanized, anti-PD-1 monoclonal antibody that has shown efficacy in multiple solid tumor types. Here, we report the results of an immunogenicity analysis of dostarlimab monotherapy in patients enrolled in the GARNET trial, a multicenter, open-label, single-arm phase 1 study. Overall, 477 of 478 patients (99.8%) were included in the analysis of dostarlimab antibody prevalence, and 349 out of 478 enrolled patients (73.0%) were evaluable for treatment-emergent antibodies to dostarlimab. The incidence of treatment-emergent ADAs was 2.5% at the recommended therapeutic dose (500 mg Q3W for the first 4 doses, 1000 mg Q6W until discontinuation), which is comparable to other anti-PD-(L)1 drugs. NAbs were detected in only 1.3% of patients. In the small percentage of patients who developed ADAs, there was no evidence of altered efficacy or safety of dostarlimab at the recommended dosing regimen. These findings demonstrated that treatment with dostarlimab was associated with a low risk of eliciting clinically meaningful ADAs over the course of this study, and dostarlimab is already approved by health authorities.

**Supplementary Information:**

The online version contains supplementary material available at 10.1208/s12248-021-00624-7.

## INTRODUCTION

The discovery of immune checkpoints has revolutionized cancer immunotherapy via immune system activation. The interaction between programmed cell death ligand 1 (PD-L1) and its receptor, programmed cell death 1 (PD-1), inhibits the immune response and has been shown to be crucial for self-tolerance, immune evasion, and autoimmunity avoidance (1). Monoclonal antibodies (mAbs)—such as pembrolizumab, nivolumab, cemiplimab, atezolizumab, avelumab, and durvalumab—block the interaction of PD-1/PD-L1 and remove the ability of tumors to evade the immune system (2, 3).

Dostarlimab (Jemperli; TSR-042) is an approved, humanized, anti-PD-1 mAb that competitively inhibits the PD-1 receptor by blocking ligand binding (4, 5). Dostarlimab has demonstrated clinical activity in various tumor types, including second-line mismatch repair-deficient endometrial cancer, mismatch repair-deficient pan tumors, and non-small cell lung cancer (NSCLC) (6–8).

Despite the clinical success of mAbs as highly effective immuno-oncology therapeutic agents, challenges remain with respect to their widespread clinical use in all patients. One of these is the emergence of immunogenicity, which occurs when antidrug antibodies (ADAs) form against an exogenous therapeutic (9). Often ADAs will not produce any overall clinically meaningful effects, but they have the potential to alter pharmacokinetics and impact safety or efficacy. Development of ADAs is dependent on patient-related factors (e.g., human leukocyte antigen type, immune competence, disease, concomitant medicines), dose regimen, route of administration, and critical product factors (e.g., primary sequence, T and B cell epitopes, expression system, glycosylation, aggregation, degradation, post-translational modification, formulation, and impurities) (10–13). ADAs may reduce efficacy by altering drug clearance and impact drug safety via infusion reactions, hypersensitivity reactions, anaphylaxis, and ADA-mediated diseases (14, 15). Another type of ADA, neutralizing antibodies (NAbs), reduce efficacy by disrupting target binding. Although humanization of antibodies is associated with a reduced risk of antidrug immunological responses, immunogenic responses are still observed in both partially humanized and fully humanized antibodies (16, 17).

The aim of the current study is to assess the immunogenicity of dostarlimab monotherapy at a recommended therapeutic dose in patients from the phase 1 GARNET trial (NCT02715284).

## MATERIALS AND METHODS

### Study Design

**Fig. 1:**
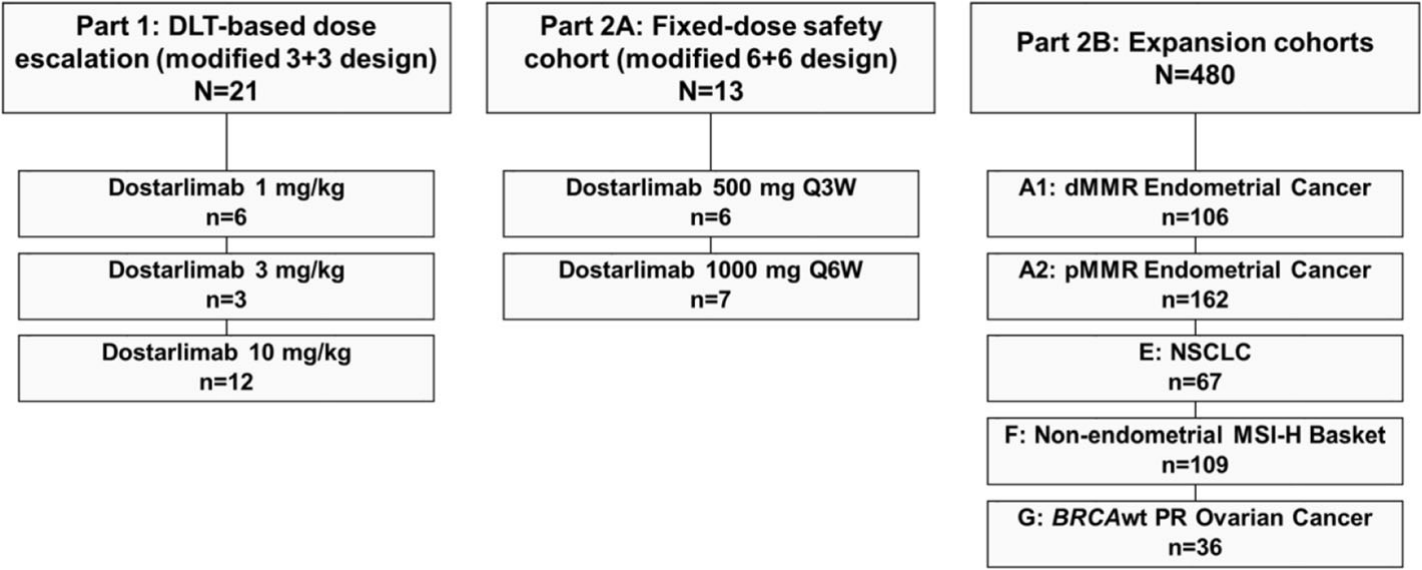
Study design. DLT, dose-limiting toxicity; dMMR, deficient mismatch repair; MSI-H, microsatellite instability high; NSCLC, non-small cell lung cancer; pMMR, proficient mismatch repair; PR, platinum-resistant; Q3W, every 3 weeks; Q6W, every 6 weeks; wt, wild-type

This multicenter, open-label, single-arm phase 1 study was conducted in two parts. Part 1 was a weight-based, dose-escalation study to determine the recommended therapeutic dose and schedule (RTD) for dostarlimab monotherapy. Part 2A was an extension of part 1 to further evaluate the safety and pharmacokinetics (PK) of dostarlimab at fixed doses based on the projection from part 1 data. Part 2B enrolled 5 expansion cohorts according to tumor type and mutation status to assess the antitumor activity and safety of dostarlimab (Fig. 1).

The trial was performed in accordance with the principles of the Declaration of Helsinki, Good Clinical Practices, and all local laws. The trial was designed by GlaxoSmithKline, the sponsor, in collaboration with the authors.

### Patient Eligibility Criteria

Eligible patients for all parts were aged at least 18 years, with histologically or cytologically proven recurrent or advanced solid tumors. For part 2B, patients had to have measurable lesion(s) per Response Evaluation Criteria in Solid Tumors (RECIST) v1.1 and meet the disease-specific criteria for each cohort. In all parts, patients who had received prior therapy with an anti-PD-1, anti-PD-L1, or anti–programmed cell death ligand 2 (PD-L2) agent were ineligible for enrollment in the study.

### Treatment

Dose escalation in part 1 followed a modified 3+3 design. If no more than 1 of 3 patients enrolled at the first dose level experienced dose-limiting toxicity (DLT), then 3 additional patients were enrolled to receive the next higher dose. However, if more than 1 of 3 patients experienced a DLT, then 3 additional patients were enrolled at the current dose. If no more than 2 of the 6 patients experienced a DLT, the current dose was considered safe, and new patients could be enrolled to receive the higher dose level. Patients received three ascending weight-based dose levels of dostarlimab—1 mg/kg, 3 mg/kg, and 10 mg/kg—administered via a 30-min intravenous (IV) infusion once every 2 weeks (Q2W). Patients in part 2A received a fixed dose of dostarlimab—either 500 mg once every 3 weeks (Q3W) or 1000 mg once every 6 weeks (Q6W)—via a 30-min IV infusion on day 1 of each cycle according to a modified 6+6 design. If no more than 1 of 6 patients enrolled at the first dose level experienced a DLT, then 6 additional patients were enrolled at the next higher dose. If more than 1 of 6 patients experienced a DLT, 6 additional patients were enrolled at the current dose. If no more than 3 of the 12 patients experienced a DLT, the current dose was considered safe, and new patients could be enrolled to receive the next higher dose level. Patients in part 2B received the RTD: dostarlimab administered intravenously at 500 mg Q3W for four doses, then 1000 mg Q6W until disease progression, treatment discontinuation due to toxicity, or patient withdrawal of consent.

### Test Sample Collection

Samples for analysis of ADAs, including NAbs, were aliquots of serum PK samples. ADAs were evaluated in parts 1 and 2A pre-dose on day 1 of each treatment cycle, post-dose at or after 96 h according to the protocol schedule, and upon treatment discontinuation at a safety follow-up visit approximately 90 days after the last dose of dostarlimab. In part 2B, ADAs were evaluated in the serum samples collected pre-dose on day 1 of cycle 1 (baseline), cycle 4 (9 weeks), cycle 5 (12 weeks), cycle 8 (30 weeks), and cycle 12 (54 weeks), and then at a safety follow-up visit approximately 90 days after the last dose of dostarlimab.

### ADA Detection Assays

The 3-tier bridging electrochemiluminescent (ECL) assay (Charles River Laboratory, Worcester, MA) for the determination of anti-dostarlimab antibodies in human serum was validated with a sensitivity in the screening assay of less than 0.4 ng/mL or 3 ng/mL for the positive control (PC) surrogate antibodies, mouse mAb, or rabbit polyclonal antibody (3.45 ng/mL as low PC for sample analysis), respectively. In the confirmatory assay, the low PC was 40 ng/mL in the presence of 405 μg/mL of dostarlimab, compared to without drug. In this bridging assay, a master mix comprising biotinylated dostarlimab (capture, 1 μg/mL) and SULFO-TAG-labeled dostarlimab (detection, 1 μg/mL) was added to each diluted (1:4 minimum required dilution) sample (with or without 405 μg/mL of dostarlimab) and incubated at 25°C for 3 h to allow for the formation of the antibody-drug complex. In the titer assay, the sample (titer control or sample) was prepared in a 3-fold dilution series (1:4, 1:12, 1:36, 1:108, 1:324, 1:972, 1:2916, and 1:8748) using AD buffer (25% NHS; 4.0 mL of NHS + 12.0 mL of low cross buffer). Subsequently, the incubated samples were transferred to a pre-blocked MSD GOLD Streptavidin plate (Meso Scale Discovery, Rockville, MD) and incubated at 25°C for 1 h to capture the drug-ADA-drug complexes. The intensity of the ECL signal generated is proportional to the amount of the captured complexes containing both biotinylated and SULFO-TAG-labeled dostarlimab. The validated screening assay is drug tolerant up to 125 μg/mL or 250 μg/mL in the presence of 100 ng/mL or 500 ng/mL of the mouse mAb PC, respectively.

Fifty-one treatment-naïve cancer sera samples were divided into 3 groups, with 17 samples in each group, and were tested on a single plate by 2 analysts in each of 3 independent assay runs using a balanced Latin square design to establish cut points. A nonparametric screening cut point factor of 1.11 at a 5% false-positive error rate was determined during validation (B2S Life Sciences, Franklin, IN). A confirmatory cut point of 40.7% inhibition with a 1.0% false-positive error rate and a titer cut point factor of 1.19 with 0.1% false-positive error rate were also computed during validation. Individual pre-dose serum samples from 467 patients enrolled in the study were used to estimate the in-study screening cut point factor. A parametric 90% lower confidence limit (LCL) screening cut point factor of 1.24 was determined for application in the tier 1 screening assays to identify serum samples that were potentially positive for the presence of ADAs to dostarlimab.

### NAb Detection Assay

The competitive ligand-binding assay (Frontage Laboratory, Exton, PA), with a sensitivity of 476.5 ng/mL and drug tolerance (DT) of at least 250 μg/mL, was validated to evaluate the neutralizing activity of anti-dostarlimab antibodies. In this assay, the anti-dostarlimab NAb (mouse monoclonal anti-idiotype antibody, the same clone as mAb PC for the 3-tier ADA assay) was incubated with dostarlimab to form an antibody-drug complex and mimic a scenario in which a neutralizing ADA is present. This mixture was incubated with biotinylated PD-1, which binds to free dostarlimab, and then with SULFO-TAG-labeled PD-L1, which binds to free PD-1. The reaction mixture was then incubated on an MSD GOLD Streptavidin plate, where the bound and free forms of the biotinylated PD-1 were captured. The intensity of the ECL signal produced is proportional to the amount of captured SULFO-TAG-labeled PD-L1. A greater amount of NAb will yield a greater amount of PD-L1 bound to PD-1 on the plate, resulting in an increased ECL signal. Sixty individual human cancer sera samples spiked with 250 μg/mL of dostarlimab underwent the drug removal process and were screened to determine the assay cut point factor. The cut point factor was determined to be 1.18, with a 1.0% false-positive rate during validation.

### Drug Concentration Detection

To assign the categories for the determined ADA results, time-matched drug concentrations must be determined. The method utilized an enzyme-linked immunosorbent assay (ELISA; Charles River Laboratory, Worcester, MA). Briefly, the analysis plate was coated with human PD-1 extracellular domain-mIgG2aFc. The samples and a biotinylated anti-human immunoglobulin (Ig) G4 antibody were added. A blue-colored readout from a plate reader at 450 nm was generated through the addition of streptavidin-horseradish peroxidase followed by 3,3’,5,5’-tetramethylbenzidine, which was proportional to the dostarlimab concentration in the serum samples. The validated assay range was 32.0 to 814 ng/mL.

### CMC Process and Product Control

Dostarlimab is a humanized mAb produced by recombinant DNA technology in a mammalian expression system using a stable Chinese hamster ovary (CHO) cell line. The chemistry manufacturing and controls (CMC) process for both drug substance and drug product was monitored to ensure that the critical quality attributes (CQAs) met quality criteria and the CMC process was stable. Table I summarizes the CQAs with associated analytical methods and impacts.

**Table I:** Methods Summary for Attributes Related to Immunogenicity

CQAs	Impact	Analytical method
Osmolality	Safety, immunogenicity	USP<785>, Ph. Eur. 2.2.35 Measurement of the freezing point depression of the solution
pH	Efficacy, safety, immunogenicity	Ph. Eur. 2.2.3, USP<791> Measured by pH meter
Appearance	Efficacy, safety, immunogenicity	Color: Ph. Eur. 2.2.2 Visual assessment Clarity: Ph. Eur. 2.2.1 Opalescence Measured by turbidimeter
Subvisible particles	Efficacy, safety, immunogenicity	USP<788>, Ph. Eur. 2.9.19 Light obscuration particle count test
Aspartate isomerization (CDR)	Efficacy, safety	Characterization peptide mapping Digested by Lys-C, separated and detected by reverse phase HPLC coupled with MS
Aggregation	Efficacy, safety	SE-HPLC
Degradation/fragmentation	Efficacy, safety	Reduced CE-SDS Non-reduced CE-SDS
Host cell DNA	Safety	qPCR
HCP	Safety and immunogenicity	CHO HCP ELISA
Protein A leachate	Safety	Protein A ELISA
Viruses	Safety	*In vitro* and *in vivo* adventitious virus testing Detection of MVM DNA by qPCR
Microorganisms	Safety	USP<61> and Ph. Eur. 2.6.12 Membrane filtration bioburden method
Bacterial endotoxin	Safety	USP <85> and Ph. Eur. 2.6.14 Kinetic turbidimetric LAL method for DS Kinetic chromogenic LAL method for DP

*CDR*, complementarity-determining regions; *CE-SDS*, capillary electrophoresis sodium dodecyl sulfate; *CHO*, Chinese hamster ovary; *CQA*, critical quality attributes; *DP*, drug product; *DS*, drug substrate; *ELISA*, enzyme-linked immunosorbent assay; *HCP*, host cell protein; *LAL*, limulus amebocyte lysate; *MS*, mass spectrometer; *MVM*, minute virus of mice; *qPCR*, quantitative polymerase chain reaction; *SE-HPLC*, size-exclusion high-performance liquid chromatography; *USP*, United States Pharmacopeia

### ADA Analyses

The set for ADA analyses included all patients who received at least one dose of the study drug with any number of serum samples as described henceforth. The ADA population is defined as patients who received at least one dose of the study drug and who provided a pretreatment serum sample and at least one post-treatment ADA serum sample at or after 96 h.

**Fig. 2:**
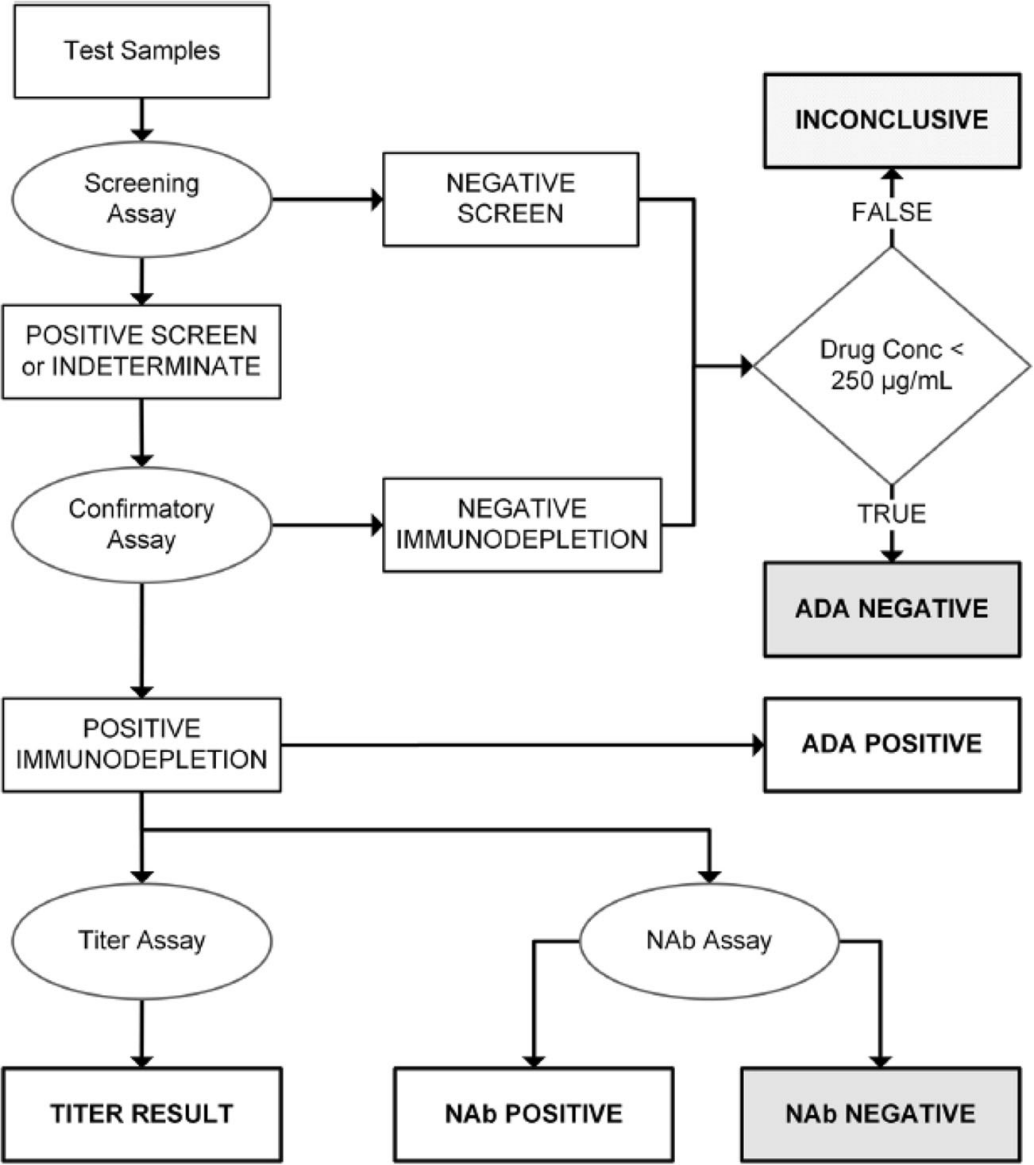
Sample testing scheme. ADA, antidrug antibody; Conc, concentration; NAb, neutralizing antibody

A 3-tiered testing paradigm was implemented to analyze serum samples for the presence of reactive antibodies to dostarlimab. A diagram of the sample testing scheme is shown in Fig. 2. Samples were tested initially in a tier 1 screening assay in which samples were classified as potentially positive, indeterminate, or negative screen for the presence of dostarlimab-reactive antibodies. Samples with a positive or indeterminate result from tier 1 screening were evaluated subsequently in tier 2 confirmatory assay using dostarlimab as the competing agent. Samples that demonstrated a percentage inhibition greater than the tier 2 cut point were classified as being positive for the presence of reactive anti-dostarlimab antibodies, whereas samples that failed to generate a percentage inhibition above the tier 2 confirmatory cut point were classified as negative immunodepletion.

Samples that were classified as negative screen or negative immunodepletion were examined further to be categorized through the concentration of dostarlimab in the sample. If the dostarlimab concentration was less than or equal to the DT of the assay (250 μg/mL), the sample was reported as ADA negative. When the dostarlimab concentration was higher than the DT of 250 μg/mL, the samples were classified as inconclusive.

Samples that were positive in tier 2 testing were submitted for further evaluation in a tier 3 titer assay to provide a quasi-quantitative estimate of the amount of antibody present. Tier 2 positive serum samples were also investigated for the presence of NAbs.

Definitions related to samples and patient testing are listed in Table II and Table S1.

**Table II:** ADA Response Categories

ADA response category	Baseline status	Post-dose status	Treatment-emergent ADA response?
Treatment-induced	Negative	Positive	Yes
Treatment-induced negative	Negative	Negative	No
Treatment-boosted	Positive	Positive, titer ≥9-fold baseline titer	Yes
Treatment-unaffected	Positive	Negative or positive with titer <9-fold baseline titer	No
ADA inconclusive	N/A	Not treatment-induced or treatment-boosted and has one or more inconclusive sample results	N/A

*ADA*, antidrug antibody; *N/A*, not applicable

### Immunogenicity and Dostarlimab PK

The impact of ADA status on dostarlimab PK was assessed in pre-dose concentration-time profiles through graphical comparison of general statistical analysis stratified by ADA/NAb status.

### Immunogenicity and Dostarlimab Safety

Dostarlimab safety was examined for all parts of the study. Adverse events (AEs) were coded according to the Medical Dictionary for Regulatory Activities (MedDRA) version 20.0 and graded according to the National Cancer Institute Common Terminology Criteria for Adverse Events version 4.03. Selected AE categories included any AEs, serious AEs (SAEs), grade 3 or higher AEs, immune-related (ir) AEs, AEs leading to study treatment interruption, AEs leading to withdrawal of study treatment, AEs with outcome of death, infusion-related reactions (MedDRA preferred term, occurring within 24 h of dose administration), and hypersensitivity (MedDRA preferred term, occurring more than 24 h after dose administration). The number and percentage of patients in each AE category were examined by patient ADA category.

### Immunogenicity and Dostarlimab Efficacy

A selected subset of efficacy measures was evaluated with respect to immunogenicity for patients enrolled in part 2B from cohorts A1 + A2 (endometrial cancer [EC]), F (mismatch repair-deficient [dMMR] pan tumors), and dMMR tumors (EC and non-EC). Specifically, objective response rate (ORR) and duration of response (DOR), as assessed by blinded independent central review using RECIST v1.1, were measured for patients by cohort and ADA category.

## RESULTS

### Patient Disposition

At the time of analysis, 477 of 478 patients (99.8%) had at least one immunogenicity sample result and were included in the analysis of prevalence: 21 from part 1, 6 from part 2A whose dostarlimab dosing schedule was Q3W, 7 from part 2A whose dosing schedule was Q6W, and 443 from part 2B. Out of the 444 total patients from part 2B, 1 had no immunogenicity results, 3 had no baseline results, and 126 had no post-baseline results. Of the 478 enrolled patients, 349 (73.0%) were evaluable for treatment-emergent antibodies to dostarlimab.

### Study Drug

The CMC process is monitored and controlled through CQAs, and batch-testing results of 11 drug substances and 17 drug products met all CQA acceptance criteria. Batch analysis and characterization studies demonstrated process consistency and no impact on potency. Overall, the data confirmed that the dostarlimab drug substance and drug product consistently present desired product quality, with very low impurity levels, thus at low risk to elicit immunogenicity.

### Prevalence of Antibodies to Dostarlimab

ADA prevalence was evaluated for all available patients at each visit by study part, cohort, and dose group. Prevalence is the proportion (%) of study patients who are positive for ADAs at a specified time point relative to the total number of patients with samples at that time point, regardless of the baseline status of patients. The prevalence of pre-existing antibodies to dostarlimab at study enrollment prior to dose administration (baseline) was 16.5%. No baseline antibodies were observed in patients in part 1, but the prevalences of ADAs at baseline were 16.7%, 14.3%, and 17.3% in parts 2A Q3W, 2A Q6W, and 2B, respectively. In part 2B, the prevalences were 5.1% at cycle 4, 5.2% at cycle 5, 4.6% at cycle 8, 0.0% at cycle 12, and 2.9% at safety follow-up (90 ± 7 days).

### Incidence of Treatment-Emergent ADAs and NAbs

The number of patients with treatment-emergent ADAs is shown by study part (parts 1, 2A, and 2B) and by cohort (EC, NSCLC, and dMMR pan tumors) for part 2B in Table III.

**Table III:** Incidence of Patients with Treatment-Emergent Anti-dostarlimab Antibodies Post-baseline by Part and Cohort

		*n*	Treatment-induced ADA	Treatment-boosted ADA	Treatment-emergent ADA^a^	ADA inconclusive
**Part,** ***n*** **(%)**						
Part 1		21	2 (9.5)	0	2 (9.5)	4 (19.0)
Part 2 – Q3W		6	0	1 (16.7)	1 (16.7)	1 (16.7)
Part 2A – Q6W		7	1 (14.3)	1 (14.3)	2 (28.6)	0
Part 2B		315	6 (1.9)	2 (0.6)	8 (2.5)	1 (0.3)
Total		349	9 (2.6)	4 (1.1)	13 (3.7)	6 (1.7)
**Part 2B cohort,** ***n*** **(%)**						
EC	dMMR	78	2 (2.6)	0	2 (2.6)	1 (1.3)
	pMMR	101	1 (1.0)	1 (1.0)	2 (2.0)	0
	MMRunk	13	0	0	0	0
E^b^		51	0	0	0	0
F^c^	dMMR^d^	67	3 (4.5)	1 (1.5)	4 (6.0)	0
	pMMR/MMRunk	5	0	0	0	0

^*a*^Treatment-induced or treatment-boosted; ^*b*^Non-small cell lung cancer; ^*c*^Non-endometrial dMMR and POLE-mut cancers (dMMR, pMMR, or MMRunk); ^*d*^Patients with POLE-mut are included

*ADA*, antidrug antibody; *dMMR*, mismatch repair-deficient; *EC*, endometrial cancer; *MMRunk*, mismatch repair unknown; *pMMR*, mismatch repair proficient; *Q3W*, every 3 weeks; *Q6W*, every 6 weeks

For the therapeutic dose and regimen (RTD), 6 of the 315 patients (1.9%) had treatment-induced ADAs and 2 patients (0.6%) had treatment-boosted ADAs, for an overall incidence of treatment-emergent ADAs of 2.5%. Of these 8 patients, the first incidence of treatment-emergent ADAs occurred at cycle 4 (*n* = 6) or cycle 8 (*n* = 1), and 1 incident occurred at a safety follow-up visit at 24 weeks. Within each cohort of part 2B, the incidence of treatment-emergent ADAs ranged from 0.0 to 6.0%, representing between 0 and 4 patients per cohort. In addition, 1 patient in the EC-dMMR cohort was classified as inconclusive with respect to treatment-emergent ADAs.

At the time of analysis, 13 patients (3.7%) had positive samples for treatment-emergent ADAs. In addition, the majority of patients had a titer of 1:36 or lower. For patients with treatment-induced ADAs (*n* = 9), the maximum observed titer was 1:36 (3 patients), with most patients having a maximum observed titer of 1:12 or lower. In the patients with treatment-boosted ADAs (*n* = 4), the maximum titer was 1:972, observed in a single sample from 1 patient, whereas the other 3 patients had maximum titer values of 1:108 (1 patient) or 1:36 (2 patients).

The rate of inconclusive patients at the RTD was 1.7% when the assay with 250 μg/mL of DT (500 ng/mL mouse mAb) was implemented; the inconclusive rate increased to 15.5% when the assay with 125 μg/mL of DT (100 ng/mL mouse mAb) was implemented. However, the incidence of treatment-emergent ADAs remained the same.

The confirmed ADA-positive samples were further evaluated for NAbs. Of the patients with treatment-emergent ADAs (13 total from all parts), 7 (2.0%) tested positive for NAbs at one or more time points, with 4 of these patients (1.3%) in part 2B. In addition, 33 patients were categorized as having treatment-unaffected ADAs (pre-existing ADAs with no meaningful increase in titer), who were positive for NAbs at one or more time points during the study.

### Persistent ADA Response

Patients with treatment-induced antibodies to dostarlimab are classified as having transient or persistent ADAs. The predefined criteria used to support the classification of patient antibody responses as being either transient or persistent are described in Table S1 (18). Overall, 8 patients (2.3% of evaluable patients) had a persistent ADA response and 1 patient (0.3%) had a transient ADA response. In the patients with persistent antibodies to dostarlimab, most (7 out of 8) were classified as persistent because of either a positive last sample (criterion P2) or a positive sample less than 16 weeks before a negative last sample (criterion P3), and only 1 patient was persistently positive at two or more time points separated by more than 16 weeks (criterion P1).

### ADA and PK

**Fig. 3:**
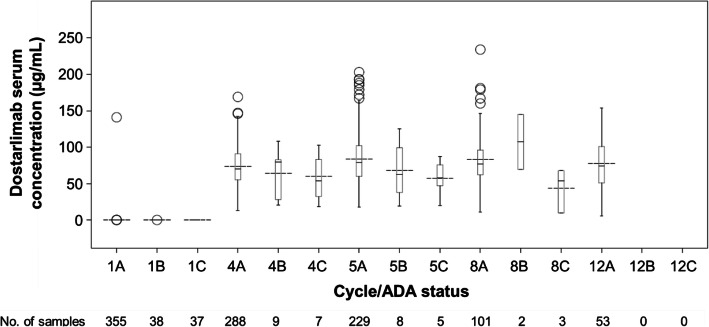
Boxplot of dostarlimab serum pre-dose concentrations for part 2B across cycles stratified by ADA/NAb status on linear scale. Boxes present median, 25% quartile, and 75% quartile. Whiskers are minimum and maximum excluding outliers (i.e., values outside of 1.5x of +/− interquartile range). Outliers are presented by symbols and mean is shown as dashed line. “*n*” is the number of observations with matched pre-dose and immunogenicity results. A represents ADA−, B represents ADA+/NAb−, and C represents ADA+/NAb+. All patients may not have reached later cycles at the time of data cutoff. ADA, antidrug antibody; NAb, neutralizing antibody.

In part 1, part 2A, and part 2B, 474 patients dosed with dostarlimab with baseline ADA results and a concentration result pair were included in the immunogenicity analysis, out of which 440 patients were from part 2B. Immunogenicity did not appear to impact PK in by-subject plots (data not shown). Box plots of dostarlimab pre-dose trough concentrations in part 2B by ADA/NAb status are presented on a linear scale in Fig. 3. Generally, when comparing the distributions of pre-dose concentrations by the ADA or NAb status, limited impact of ADAs on pre-dose concentrations was observed. This was deemed not clinically relevant, despite a trend of lower mean concentrations in ADA-positive records, regardless of NAb status. The impact of ADAs/NAbs on PK was assessed in the dostarlimab population PK analysis, which will be published separately.

### Adverse Events

The frequency of AEs is summarized in Table IV by AE category and ADA status for patients treated with the RTD as well as for ADA populations. One patient was excluded from the comparison due to inconclusive ADA status. For all 7 AE categories, there were no observed differences between treatment-unaffected ADAs, treatment-induced negative ADAs, NAb positive, or overall safety populations. This held true for treatment-emergent ADAs in all AE categories except irAEs, where the rate was 62.5% compared with approximately 40% (34.26 to 43.24%) for all other ADA categories and safety populations. However, when including data from all parts (parts 1, 2A, and 2B) of the study, the rate of treatment-emergent ADA irAEs (5 out of 13 patients, 38.5%) was similar to negative ADA populations (108 out of 330 patients or 32.7%). Infusion reactions and hypersensitivity were low for dostarlimab. No infusion reactions were observed in the treatment-emergent ADA population. In the ADA population excluding those with treatment-emergent ADAs, 1.5% experienced an infusion reaction. One in 13 from the treatment-emergent ADA population had hypersensitivity, with none from other ADA sub-populations.

**Table IV.** Adverse Events by Patient ADA and NAb Status (Antidrug Antibody Population)

Variable, *n* (%)	Treatment-unaffected ADA, *n* = 55	Treatment-emergent ADA, *n* = 8	Treatment-induced negative ADA, *n* = 251	NAb positive, *n* = 37	Inconclusive ADA, *n* = 1	Safety population, *n* = 444
Any AEs	55 (100.0)	8 (100.0)	244 (97.21)	37 (100.0)	1 (100.0)	428 (96.40)
Any grade ≥3 AEs	23 (41.82)	2 (25.00)	108 (43.03)	19 (51.35)	1 (100.0)	212 (47.75)
Any irAE	20 (36.36)	5 (62.50)	86 (34.26)	16 (43.24)	1 (100.0)	145 (32.66)
Any SAEs	17 (30.91)	1 (12.50)	82 (32.67)	12 (32.43)	1 (100.0)	166 (37.39)
Any AE leading to study treatment interruption	14 (25.45)	1 (12.50)	54 (21.51)	10 (27.03)	0	98 (22.07)
Any AEs leading to withdrawal of study treatment	3 (5.45)	1 (12.50)	13 (5.18)	3 (8.11)	1 (100.0)	33 (7.43)
Any AE with outcome of death	2 (3.64)	0	4 (1.59)	2 (5.41)	1 (100.0)	16 (3.60)

Safety population: all patients who receive any amount of study drug in part 2B

*ADA*, antidrug antibody; *AE*, adverse event; *ir*, immune-related; *NAb*, neutralizing antibody; *SAE*, serious adverse event

### Efficacy Measures

NAb production may impact treatment efficacy and is a common regulatory concern. Selected efficacy endpoints for part 2B EC and pan-tumor cohorts are summarized in Table V by cohort and NAb status. Twenty-one patients were NAb positive in the EC cohort; of these patients, 7 (33.33%) were classified as having complete or partial response (ORR), with 4 patients having a duration of response (DOR) of at least 6 months. Both ORR and DOR results were comparable to NAb-negative patient results. In pan-tumor dMMR patients with or without EC, efficacy in the NAb-positive population was comparable or better than that of NAb-negative patients, indicating that immunogenicity did not impact efficacy.

**Table V.** Summary of Efficacy Endpoints by NAb Status (Antidrug Antibody Population and Efficacy Population—Part 2B)

Variable	Total NAb positive, *n* = 24	Total NAb negative, *n* = 152	ADA inconclusive, *n* = 1
EC total			
N-trt	21	114	1
ORR, *n* (%)	7 (33.33)	34 (29.82)	1 (100.0)
DOR, *n* (%)			
<6 months	3 (14.29)	10 (8.77)	0
≥6 months	4 (19.05)	24 (21.05)	1 (100.0)
Cohort F total			
N-trt	3	38	0
ORR, *n* (%)	3 (100.0)	17 (44.74)	0
DOR, *n* (%)			
<6 months	2 (66.67)	10 (26.32)	0
≥6 months	1 (33.33)	7 (18.42)	0
Total dMMR			
N-trt	11	87	1
ORR, *n* (%)	9 (81.82)	40 (45.98)	1 (100.0)
DOR, *n* (%)			
<6 months	5 (45.45)	15 (17.24)	0
≥6 months	4 (36.36)	25 (28.74)	1 (100.0)

Efficacy population: All patients in safety population with measurable disease at baseline (defined as the existence of at least one target lesion) who have MMR status based on IHC testing (local or central) for dMMR and PCR or NGS testing (local or central) for MSS and MMR unknown. POLE-mutation in F cohort is included in dMMR

Percentages are based on n/N-trt; *ADA*, antidrug antibody; *dMMR*, mismatch repair-deficient; *DOR*, duration of response; *EC*, endometrial cancer; *n*, number of patients with endpoint; *NAb*, neutralizing antibody; *N-trt*, number of patients in group; *ORR*, objective response rate

## DISCUSSION

A 3-tier ADA and NAb assay was established for immunogenicity assessment in cancer patients who received dostarlimab. Of the 477 total patients who had serum samples available, the majority (93%) were collected from patients enrolled in part 2B (at RTD) of the GARNET study.

**Fig. 4:**
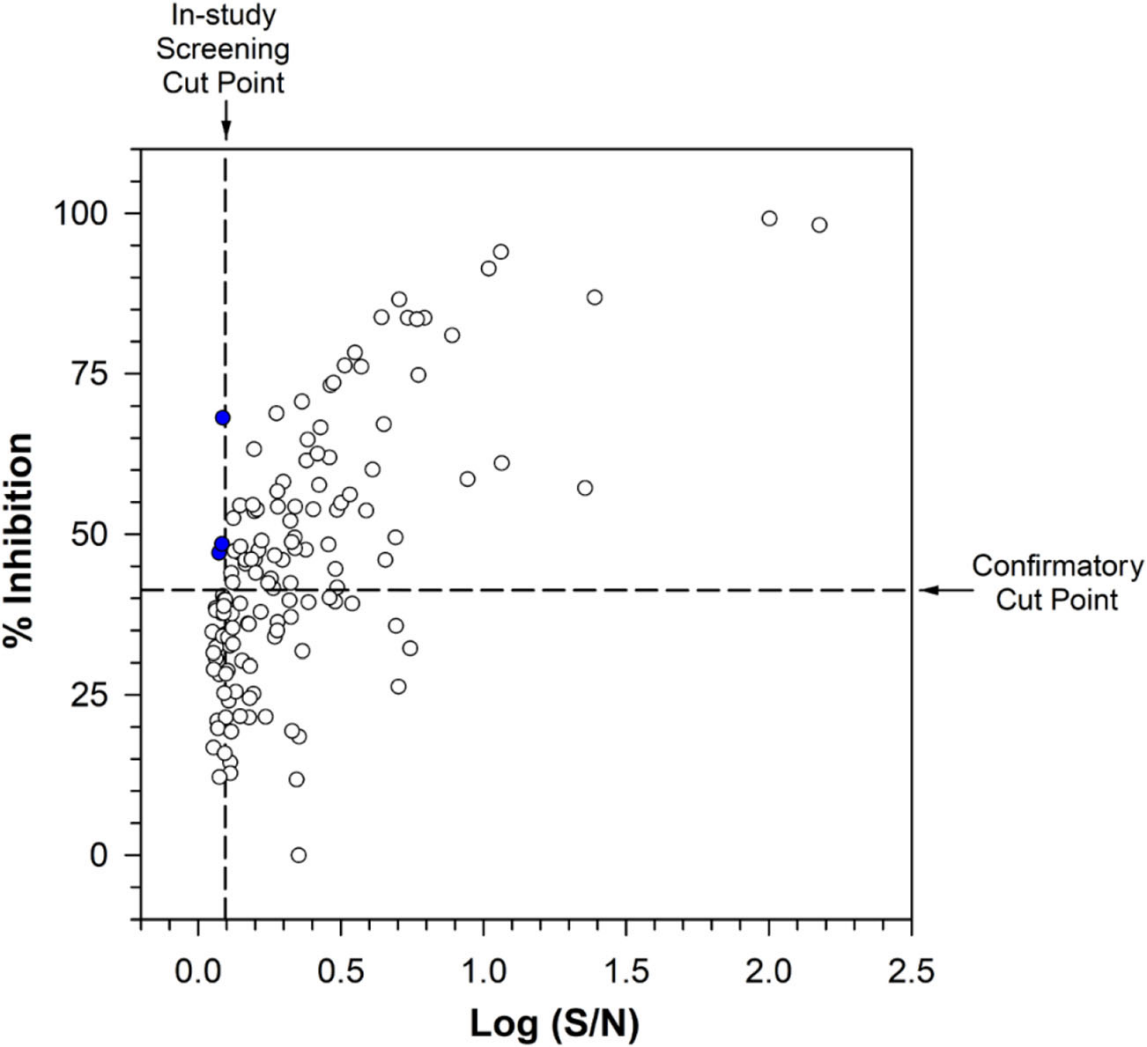
Scatterplot of percent inhibition *vs*. log S/N ratio – individual subject samples at baseline for subjects in part 2B with both screening and confirmatory results. The vertical reference line of 0.093 is the in-study 95% nonparametric screening cut point, which corresponds to an S/N ratio value of 1.24. The horizontal reference line of 40.7 is the 99% nonparametric validation confirmatory cut point. Solid red points were screened positive based on the validation screening cut point but screened negative based on the in-study cut point

The prevalence of ADA-positive samples at baseline was 16.5% and fell to a lower level (range, 0.0 to 5.2%) at cycles 4, 5, 8, 12, and safety follow-up. The small proportion of antibody-negative samples that were classified as inconclusive (2.4% for all parts) and the detailed examination of ADA results do not suggest that the observed decrease from high prevalence of pre-existing ADAs was solely due to drug interference in the ADA assay. There were 19 samples from 9 patients that screened positive, with concentrations greater than or equal to the assay tolerance level of 125 μg/mL (at 100 ng/mL sensitivity). Four of these 19 samples had dostarlimab concentrations (222, 229, 232, and 287 μg/mL) close to 250 μg/mL of drug tolerance (at 500 ng/mL sensitivity). Four of the 9 aforementioned patients had samples with dostarlimab concentrations greater than or equal to 125 μg/mL and were confirmed positive for ADAs, including one with the highest measured drug concentration of 171 μg/mL (early-onset ADAs at cycle 1 day 5). Further evaluation demonstrated that drug concentrations did not impact titer determination or signal detection for the validated 3-tier ADA method (data on file). To evaluate if the assay cut point established in validation was appropriate, an in-study cut point of 1.24, which is higher than the originally calculated validation cut point of 1.11, was determined. If the in-study cut point had been used in lieu of the validation cut point during the analysis of patient samples, fewer samples would have been classified as being potentially positive for reactive antibodies. Upon retrospective application of the in-study cut point, the number of samples affected by application of the in-study cut point is small and results in fewer samples being identified as ADA positive. This change is illustrated in Fig. 4, where three baseline samples from subjects in part 2B that were confirmed positive upon application of the original validation screening cut point screened as negative with the use of the in-study cut point. The overall classification changed only modestly, with four samples (three at baseline and one at safety follow-up) from 4 different patients in part 2B that originally confirmed as being ADA positive now being categorized as ADA negative. The changes include 1 patient categorized as negative at baseline who did not have any post-baseline samples, 1 patient changed from treatment-induced ADA to treatment-induced negative, and 2 patients changed from treatment-unaffected ADA to treatment-induced negative ADA. Based on this retrospective analysis, no significant effect was found on the overall interpretation of the immunogenicity findings for this study, and pre-existing prevalence was slightly changed from 17.3 to 16.6% for part 2B.

The high rate of pre-existing antibodies (more than 15%) in ADA assays is more likely from unidentified factor(s). The high sensitivity of the assay without drug present (<0.4 ng/mL) may allow for increased detection of low concentrations of ADAs. Target interference may be a concern. However, PD-1 is unlikely the cause since it cannot form dimers, which is required for the bridging assay format (19). Other underlying factors, such as cross-reactivity (19, 20) and existence of heterophile antibodies (dostarlimab was derived from a mouse mAb with 4.1% mouse/95.9% human) in human sera (21, 22), may also contribute to the prevalence found at baseline. However, there was no evidence of an increase in the levels of these baseline antibodies on treatment with dostarlimab. In general, titers were low for pre-existing ADAs and ADAs during treatment.

Figure 3 shows the ADA/NAb incidence during the treatment process. The ratios of ADA−:ADA+/NAb−:ADA+/NAb+ when normalized to 100 for ADA− for each cycle are as follows: cycle 1 pre-dose: 100:10.7:10.4; cycle 4 pre-dose: 100:3.13:2.43; cycle 5 pre-dose: 100:3.49:2.18; cycle 8 pre-dose: 100:1.98:2.97; cycle 12 pre-dose: 100:0:0. Unlike cycle 1, which had high pre-existing ADA+/NAb− and ADA+/NAb+, as discussed above, cycles 4, 5, and 8 had fairly consistent ADA+/NAb− and ADA+/NAb+. There was no observed ADA+/NAb− or ADA+/NAb+ at cycle 12. This finding may be due to small sample size and low incidence of ADA+/NAb− or ADA+/NAb+ associated with dostarlimab. Potential contributions of ADA status to lack of survival will be further investigated in the ongoing study and exposure–overall survival analysis when data are mature.

The dostarlimab ADA incidence rate in this study (2.5% at RTD) was similar to other anti-PD-(L)1 drugs, as shown in their prescribing information (pembrolizumab combined all dose and regimen, 2.1%; nivolumab at 3 mg/kg Q2W, 11.2%; cemiplimab, 1.3%; avelumab at 10 mg/kg Q2W, 4.1%; durvalumab at 10 mg/kg Q2W, 2.9%) with the exception of atezolizumab (1200 mg Q3W), where the ADA incidence rate ranges from 30 to 48% depending on indication (23–28). Among factors such as patient-related factors, dose regimen, route of administration, and critical product factors, target or mechanism of action may be most responsible for the observed ADA incidence. An example of tumor necrosis factor (TNF)-α inhibitors (29), infliximab is a chimeric human/murine IgG1 mAb that can potentially elicit more immunogenicity, and adalimumab is the first fully human IgG1 mAb that is expected to have less immunogenicity. When administered as monotherapy, infliximab and adalimumab have similar ADA incidence rates (30, 31). As stated in the immunogenicity labels of biologics, direct comparison between two mAbs may be misleading because the detection of antibody formation is highly dependent on the sensitivity, drug tolerance, and specificity of the assay (30–32). The observed incidence of antibody (including neutralizing antibody) positivity in an assay may be influenced by several other factors as well, including sample handling, timing of sample collection, and concomitant medications. Nevertheless, based on the overall trial data for these two products—regardless of methods, patient population, product-related factors, etc.—ADAs were highly prevalent in the treated patients (33), indicating that the target may play an important role in ADA incidence for well-designed mAbs.

Another large analysis evaluated 40 immunomodulatory (IMD) and 19 non-IMD agents in oncological or non-oncological indications (34). IMD agents include all drugs that may directly or indirectly modulate immune cells. The likelihood of a high ADA rate appears to be greater for IMD mAb agents (IMD, 18%; non-IMD, 11%). For IMD agents, B cell–depleting mAbs were associated with a low ADA rate (<15%) based on the mechanism of action. Higher rates were observed for targets expressed on T cell or myeloid antigen-presenting cells (dendritic cells, macrophages, and monocytes combined). However, it is possible to have a low ADA incidence rate, as exemplified in anti-PD-1 drugs, indicating that the target may be a driving factor. Preclinical tools, i.e., both *in silico* and *in vitro* assays with associated modeling for prediction of ADAs, can be used to select the mAb with lower immunogenicity risk to avoid the drug design–related risk for immunogenicity (29).

The method development for this ADA evaluation was initiated in 2015. At the time of development, the recommended method sensitivity was 250 to 500 ng/mL. In a 2017 white paper (35), a sensitivity of at least 100 ng/mL was recommended, although a limit of sensitivity greater than 100 ng/mL may be acceptable depending on risk and prior knowledge. In 2019, the FDA released new guidance, and 100-ng/mL sensitivity is recommended with a philosophy similar to the 2017 white paper (32).

For dostarlimab, the 3-tier ADA method, with a sensitivity of 500 ng/mL and DT of 250 μg/mL, was selected based on prescribing information for both pembrolizumab and nivolumab, which indicated low incidence and no ADA impact to PK, efficacy, or safety. In addition, the DT of 250 μg/mL was higher than the trough concentrations from nearly every patient treated at RTD (data on file); thus, the inconclusive rate was 1.7% of patients. When results were re-evaluated using the current required sensitivity of 100 ng/mL with 125 μg/mL of DT, the inconclusive rate increased to 15.5% of patients. However, the treatment-emergent ADA rate remained at 2.5%.

A competitive ligand binding assay, based on the NAbs mechanism of action, was initiated in parallel with the 3-tier ADA assay. Once the DT was established at 250 μg/mL for a sensitivity of 500 ng/mL in the 3-tier ADA assay, the NAb method was developed to achieve the full potential to catch NAb after going through the 3-tier ADA assay. The same clone of mouse mAb was used as PC in the 3-tier ADA and NAb assays for the sensitivity evaluation. The NAb assay had similar sensitivity as the 3-tier ADA assay, about 500 ng/mL, at the spiked drug concentration of 250 μg/mL. The titer distributions were similar in both the NAb-positive and NAb-negative samples. The medians (minimum, maximum) were 12 (1, 972) for NAb-positive samples and 12 (1, 972) for NAb-negative samples; the respective geometric means of the titers were 13 (95% CI, 9–18) and 12 (95% CI, 7–16). Thus, no association was seen between higher titer values and the presence of NAbs. In addition, based on the 3-tier ADA titer results with treatment-emergent ADAs, the majority of patients had a titer of 1:36 or lower. Overall, the ADA rate for dostarlimab was low with low titer, regardless of which sensitivity level was used.

The major concern for ADA and NAb is the impact on PK, safety, and efficacy. Consistent with other PD-1 checkpoint inhibitors (21–26, 29), there was no evidence that the presence of ADA or NAb resulted in a significantly altered PK profile or increased safety concerns including infusion-related reactions.

The major concern for NAbs is their impact on efficacy, which, based on our immunogenicity analysis, appears to be unlikely (Table V). A comprehensive evaluation of ADA impact on safety indicated that the immunogenicity of dostarlimab was also not a concern (Table IV). In the ADA population (part 2B), the rate of irAE was slightly higher in the treatment-emergent population than that in other ADA sub-populations and the safety population. When evaluating the treatment-emergent ADA population from all three study parts (1, 2A, and 2B) of the study, the irAE rate was comparable between treatment-emergent ADA and negative ADA populations. The pooled data more appropriately characterizes the relationship as current knowledge indicates that the development of irAEs for anti-PD-1/PD-L1 drugs was unrelated to the dose (31). Overall, the percentage of AEs in each category did not appear to be related to baseline ADA status, ADA status (positive or negative), or NAb status (positive or negative).

Additionally, the trough concentrations at the RTD were above the lowest concentration required for full receptor occupancy in both peripheral and tumor sites during the treatment process (in press). When taken together, this information shows that dostarlimab presents low to no risk of clinically associated ADA/NAb impact on efficacy or safety at the detecting sensitivity of 500 ng/mL.

All protein therapeutics are potentially immunogenic, and product-specific impact on immunogenicity is assessed using a risk assessment strategy during product development. Product origin is an important factor that can influence immunogenicity. Because dostarlimab is humanized (4.1% mouse and 95.9% human), it is anticipated to have a low immunogenicity risk profile, similar to other human/humanized mAbs.

Determination of dostarlimab’s drug substance and drug product CQAs has also taken into consideration the potential immunogenicity impact, including osmolality, pH, and appearance; process-related impurities (host cell protein (HCP), host cell DNA, protein A leachate, and subvisible particles); adventitious agents (microorganisms, bacterial endotoxin, viruses); and product-related impurities (aggregates, degradants/fragments, post-translational modifications). These CQAs are monitored with a comprehensive control strategy: raw material sourcing and testing; cell banking and testing; controlling the introduction of impurities and adventitious agents into the process via environmental, equipment, and process controls; leachable/extractable studies; small-scale impurity spiking studies; small-scale viral clearance validation studies; process and product characterization; validation of process consistency and impurity removal at a commercial scale (drug substance and drug product process performance campaigns); in-process and batch-release tests; and product stability testing. Aggregated proteins, as an example of control strategy, have higher immunogenic potential through the impact of binding to the lymph-1 receptor, Fcγ, Fc Gamma receptors and FcRn receptors compared with the monomer (36, 37). While smaller aggregates (dimers and trimers) appear inefficient in inducing immune responses, large multimers (molecular weight exceeds 100 kD) are efficient inducers of immune responses (36). Limiting the level of aggregate through process control with appropriate specifications will effectively lower the risk of immunogenicity.

All the factors combined confirm that dostarlimab drug substance and drug product present good process control and thus low risk to elicit immunogenicity. In addition, dostarlimab is administered intravenously, a route of administration that is associated with low immunogenic response.

## CONCLUSIONS

Considering serum ADA results to date from validated assays for ongoing clinical studies of dostarlimab used in accordance with common industry-tiered ADA testing approaches, we conclude that dostarlimab has an ADA incidence rate (2.5%) comparable to other anti-PD-(L)1 drugs, and treatment with dostarlimab elicits only a weak immune response in a small percentage of patients with cancer after one or more treatment cycles. The high product purity of dostarlimab and route of administration decrease the risk for induction of immune responses. Furthermore, there is currently no evidence regarding the impact of pre-existing ADAs or ADA formation on any safety or efficacy measures. These findings indicate that dostarlimab is a new and effective anti-PD-1 mAb with a low potential to elicit immunogenic responses.

## Supplementary Information

ESM 1 (DOCX 28 kb)

## Data Availability

Anonymized individual participant data and study documents can be requested for further research from www.clinicalstudydatarequest.com
