# Supplementary material for: An Integrated Analysis of Dostarlimab Immunogenicity
Source: AAPS J. 2021 Jul 29;23(5):96. doi: 10.1208/s12248-021-00624-7 (PMC8321970; doi:10.1208/s12248-021-00624-7)
Supplement: Supplementary file 1 — (DOCX 28 kb) [file 12248_2021_624_MOESM1_ESM.docx]

# SUPPLEMENTARY APPENDIX

## Table S1. Sample and patient testing-related definitions

| **Sample and patient testing term** | **Definition** |
| --- | --- |
| Negative screen | A sample that produces a signal response less than the plate-specific cut point (PSCP) in a tier 1 screening assay. A negative screen will be reported as NEGATIVE for the presence of reactive antibodies. |
| Positive screen | A sample that produces a signal response greater than or equal to the PSCP in a tier 1 screening assay. A positive screen will be reported as POTENTIALLY POSITIVE for reactive antibodies and submitted for testing in a tier 2 confirmation assay. |
| Indeterminate screen | In a tier 1 screening assay, no definite result is obtained for a specific sample, which is further tested in the confirmatory assay. |
| Negative immunodepletion | A sample that produces % inhibition result less than the confirmatory cut point (CCP) in a tier 2 assay. Negative immunodepletion will be reported as NEGATIVE for the presence of reactive antibodies. |
| Positive immunodepletion | A sample that produces % inhibition result greater than or equal to the confirmatory cut point (CCP) in a tier 2 assay. A positive immunodepletion will be reported as being POSITIVE for the presence of reactive antibodies and submitted for testing in a tier 3 titer assay. |
| Titer or titer value | The largest dilution factor at which the signal response value of the sample is greater than or equal to the plate-specific titer cut point. A titer value is reported as the reciprocal of the dilution factor including the MRD.  For example, a titer of 9 with a 1:4 MRD would be reported as 1:36.  A titer reported as <4 or “negative titer” will be considered as “1” for analyses. |
| ADA negative sample | A sample that is not confirmed to be positive for the presence of drug reactive antibodies. An ADA negative sample either fails to generate a positive response in a tier 1 screening assay or is positive or indeterminate in tier 1 but not positive in a tier 2 confirmatory assay. Results are reported as “negative screen” or “negative immunodepletion.” |
| ADA positive sample | A sample that is confirmed to be positive for the presence of reactive antibodies. A sample must generate a positive or indeterminate response in the tier 1 screening assay and positive in the tier 2 confirmatory assay. |
| ADA inconclusive sample | A sample that fails to be confirmed as being positive for reactive antibodies, but which is suspected to have the therapeutic agent present at a concentration that exceeds the known tolerance limit for the ADA assay (ie, drug concentration that is determined empirically to cause interference in ADA detection). In this specific assay, the drug tolerance limit is 250 µg/mL for 500 ng/mL or 125 µg/mL for 100 ng/mL positive control. |
| Baseline sample | A patient sample collected for ADA testing at study initiation or early in study execution prior to the initial administration of drug as outlined in the protocol. |
| Pre-existing ADA | Reactive antibodies that are present in a patient test sample at study initiation prior to drug administration; these are synonymous with “baseline ADA.” |
| ADA prevalence | Proportion of study patients who are positive for ADA at a specified time point relative to the number of patients with samples at that time point. |
| ADA incidence | The proportion of patients with treatment-induced or boosted ADA responses during the study. |
| Treatment-induced ADA | ADA that develops de novo following drug administration in a patient without pre‑existing ADA. |
| Treatment-boosted ADA | Reactive antibodies that are increased by a pre-defined, biologically relevant amount relative to baseline in patients who were determined to be ADA positive at baseline. For this specific study, the pre-defined amount will be ≥9x the baseline titer. |
| Treatment-emergent ADA | A patient is categorized as having treatment-emergent ADA if the patient has either treatment-induced or treatment‑boosted ADA. |
| Treatment-unaffected ADA | Patient with pre-existing (baseline) reactive antibodies who does not show a pre-defined increase (≥9x the baseline titer for this study) in ADA levels upon drug treatment. |
| ADA positive patient | A patient who has at least 1 treatment-induced or treatment-boosted ADA response at any time during the study. |
| ADA negative patient | A patient who does not have a treatment-induced or treatment-boosted ADA response at any time during the study and does not have any inconclusive results. |
| ADA inconclusive patient | A patient who:   - Does not have a treatment-induced or treatment-boosted ADA response at any time during the study AND - Has 1 or more inconclusive results |
| Transient ADA response | Transient ADA response:  T1: Treatment-induced ADA detected only at one sampling time point during the study more than 16 weeks before the last sampling time point  or  T2: Treatment-induced ADA detected at 2 or more sampling time points during the study, where the first and last ADA-positive samples (irrespective of any negative samples in between) are separated by a period less than 16 weeks, and the patient’s last sampling time point is ADA-negative. To differentiate from P3, the last positive sample must be at least 16 weeks before the last sampling time point. |
| Persistent ADA response | P1: Treatment-induced ADA incidence detected at 2 or more sampling time points during the study, where the first and last ADA positive samples (irrespective of any negative samples in between) are separated by a period of 16 weeks or longer;  or  P2: Treatment-induced ADA incidence only in the last sampling time point of the study;  or  P3: Treatment-induced ADA incidence at a sampling time point with less than 16 weeks before an ADA‑negative last sample. |
